# Differential immune transcriptomic profiles between vaccinated and resolved HCV reinfected subjects

**DOI:** 10.1371/journal.ppat.1010968

**Published:** 2022-11-15

**Authors:** Sabrina Mazouz, Eduardo Salinas, Nathalie Bédard, Ali Filali, Omar Khedr, Leo Swadling, Mohamed S. Abdel-Hakeem, Asiyah Siddique, Eleanor Barnes, Julie Bruneau, Arash Grakoui, Naglaa H. Shoukry

**Affiliations:** 1 Centre de Recherche du Centre hospitalier de l’Université de Montréal (CRCHUM), Montréal, Québec, Canada; 2 Département de microbiologie, infectiologie et immunologie, Université de Montréal, Montréal, Québec, Canada; 3 Emory University School of Medicine, Emory University, Atlanta, Georgia, United States of America; 4 Emory National Primate Research Center, Atlanta, Georgia, United States of America; 5 Division of Infection and Immunity, University College London, London, United Kingdom; 6 Nuffield Department of Medicine, University of Oxford, Oxford, United Kingdom; 7 Département de médecine familiale et de médecine d’urgence, Université de Montréal, Montréal, Québec, Canada; 8 Département de médecine, Université de Montréal, Montréal, Québec, Canada; Albany Medical College, UNITED STATES

## Abstract

Successive episodes of hepatitis C virus (HCV) infection represent a unique natural rechallenge experiment to define correlates of long-term protective immunity and inform vaccine development. We applied a systems immunology approach to characterize longitudinal changes in the peripheral blood transcriptomic signatures in eight subjects who spontaneously resolved two successive HCV infections. Furthermore, we compared these signatures with those induced by an HCV T cell-based vaccine regimen. We identified a plasma cell transcriptomic signature during early acute HCV reinfection. This signature was absent in primary infection and following HCV vaccine boost. Spontaneous resolution of HCV reinfection was associated with rapid expansion of glycoprotein E2-specifc memory B cells in three subjects and transient increase in E2-specific neutralizing antibodies in six subjects. Concurrently, there was an increase in the breadth and magnitude of HCV-specific T cells in 7 out of 8 subjects. These results suggest a cooperative role for both antibodies and T cells in clearance of HCV reinfection and support the development of next generation HCV vaccines targeting these two arms of the immune system.

## Author summary

In this study we examined the memory immune response against hepatitis C virus (HCV) upon re-exposure and reinfection followed by spontaneous clearance. This represents a model to define the key immune signatures that should be elicited by a vaccine for long-term protective immunity. We observed that antibodies as well as white blood cells (lymphocyte) responses were induced in the majority of the subjects studied suggesting their cooperative role in protection against HCV. Such a coordinated response was lacking in an experimental HCV vaccine regimen. Altogether, these data suggest that new vaccines against HCV should combine these two arms of the immune system.

## Introduction

Worldwide, 58 million individuals are chronically infected with hepatitis C virus (HCV). Around a quarter of acutely infected individuals will spontaneously clear the virus, while the majority develops persistent infection, a leading cause of liver cirrhosis and hepatocellular carcinoma [1]. Since 2014, the introduction of direct-acting antivirals (DAA) enabled the cure of chronic HCV in the majority of treated subjects (~95%) [2]. However, DAA treatments are expensive and do not protect against reinfection. Altogether, these factors urge the development of a prophylactic vaccine to efficiently and rapidly curb the HCV epidemic [3].

High risk populations like people who inject drugs (PWID) are continuously re-exposed to HCV and thus get reinfected even if they have spontaneously cleared a primary infection [4]. Spontaneous resolution of an HCV reinfection has been associated with a shorter duration and magnitude of viremia, a broadened cellular immune response and, in 60% of cases, generation of cross-reactive antibodies [5]. We have demonstrated that the expansion of polyfunctional memory CD4^+^ and CD8^+^ T cells as well as that of a more focused HCV-specific CD8^+^ T-cell receptor repertoire comprised of public clonotypes correlated with protective immunity [6–8]. Yet, deeper insights into what constitutes a potent protective immune response in real life settings is crucial for the development of a prophylactic HCV vaccine.

To date, only one HCV vaccine has reached phase II clinical trials in humans. This T-cell based vaccine strategy is comprised of the chimpanzee adenovirus 3 (ChAd3) encoding the HCV genotype 1b non-structural (NS) proteins followed by a modified vaccinia Ankara (MVA) boost (ChAd3-NS/MVA-NS). While strong CD8^+^ and CD4^+^ T cell responses were induced in healthy volunteers [9], the vaccine eventually failed to protect against persistent infection in PWID although a reduction in HCV viraemia was observed in ChAd3-NS/MVA-NS HCV vaccinees following infection [10]. The immunological mechanisms underlying the vaccine’s failure to protect against chronic infection in PWID are not yet understood.

Systems immunology represents a powerful approach for an in-depth characterization of the early transcriptomic signatures induced by viral infections in humans [11, 12]. Similarly, systems vaccinology was successfully applied to study the transcriptomic response to either licensed vaccines including Yellow Fever 17D (YF-17D) [13, 14] and Influenza [15], or candidate vaccines against HIV [16, 17]. We have previously performed longitudinal transcriptomic analysis of the immune response in the peripheral blood during acute resolving HCV infection [12]. We observed strong innate antiviral signatures early during acute infection that returned to baseline levels following viral clearance. These innate signatures were also shared with other flaviviruses such as Dengue and YF-17D [12]. However, we observed limited adaptive immune responses suggesting that they may be restricted to a small cellular subset or are compartmentalized in the liver. Transcriptomic signatures of the recall immune response upon HCV re-exposure and reinfection and whether this response can be mimicked by HCV vaccines remain unexplored.

Herein, we applied a systems immunology approach to characterize the peripheral blood transcriptomic signatures during natural cases of HCV reinfection and to compare changes relative to those previously reported using data from primary infection in the same cohort [12] and following ChAd3-NS/MVA-NS vaccination in healthy individuals [18]. Finally, we used different functional immunological approaches to study concurrently the longitudinal HCV-specific T and B cells responses during reinfection in PWID. We identified a plasma cell transcriptomic signature in viremia positive time points unique to acute HCV reinfection. This signature was not detectable during either primary infection or following HCV vaccine boost. Acute HCV reinfection and clearance was accompanied by a transient increase in anti-glycoprotein E2 antibody responses as well as increased breadth and magnitude of virus-specific T cells, suggesting a role for both arms of the adaptive immune response.

## Results and discussion

### A robust host response in the Early acute phase of reinfection in PWID

**Fig 1:**
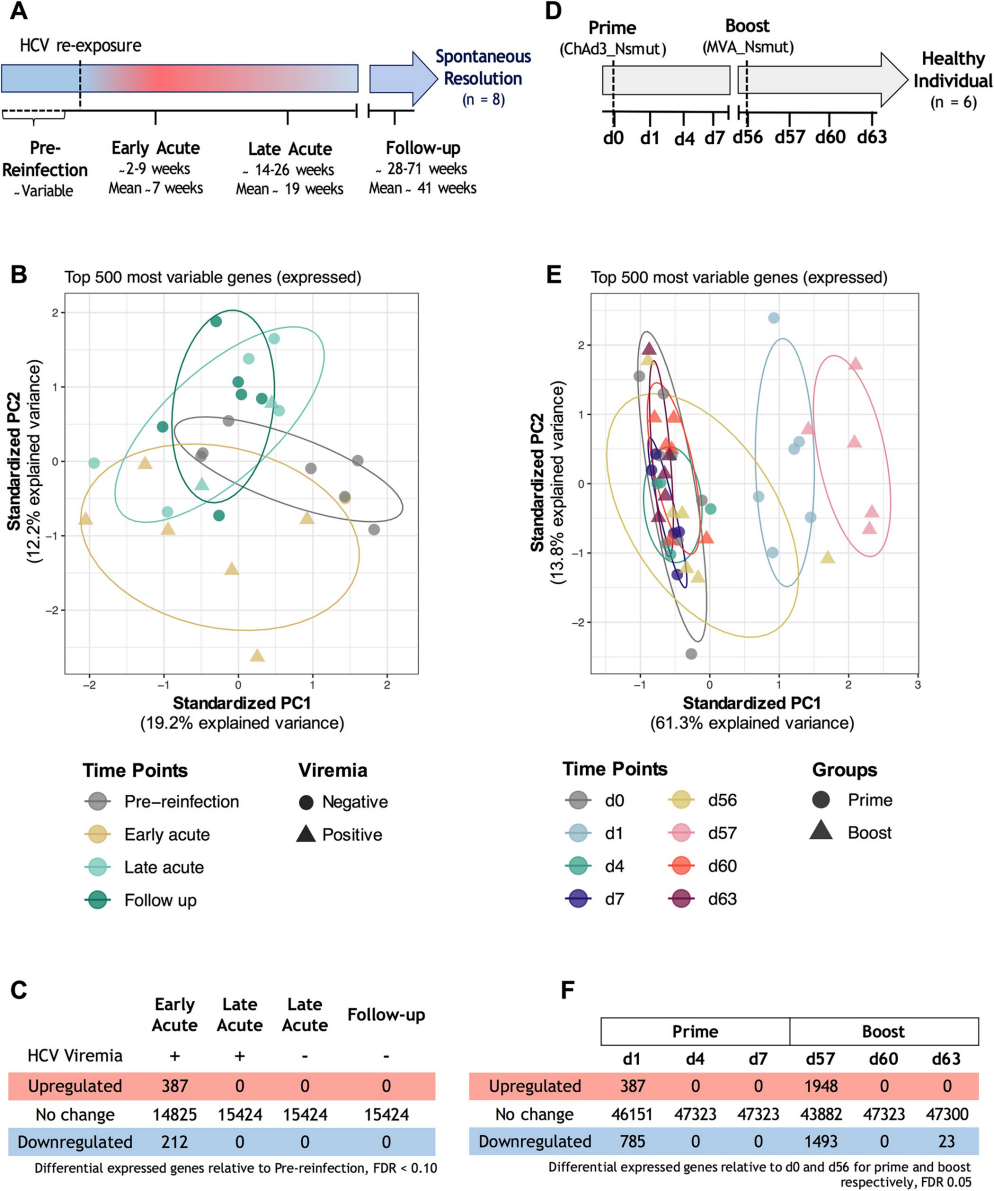
Study design and differential gene expression analysis. **(A)** Reinfection study design. **(B)** Principal component analysis (PCA) of the top 500 genes expressed in the reinfection group. As described in Materials and Methods, patient-specific gene expression variation was removed using the function removeBatchEffect. Time points and groups are indicated by different colors and shapes, respectively. The ellipses denote the core area with a confidence interval of 68% for each group. **(C)** Number of differentially expressed genes (DEGs) across all reinfection time points. Pre-reinfection time-points were used as a baseline. Genes differentially expressed are selected using a Benjamin-Hochberg adjusted *P* value < 0.10. **(D)** Vaccine study design. Vaccine data were obtained from Hartnell et al. [18]. **(E)** PCA of the top 500 genes expressed in the vaccine group. **(F)** Number of DEGs across all vaccine samples. d0 and d56 were used as a baseline for prime and boost vaccine time points, respectively. Genes differentially expressed are selected using a Benjamin-Hochberg adjusted *P* value < 0.05.

The Montreal hepatitis C cohort (HEPCO) recruits and follows PWID subjects during multiple episodes of HCV infection, representing a unique opportunity to study the immune response during acute reinfection [19]. For this study, we selected 8 subjects from the HEPCO cohort who became reinfected after spontaneously clearing a primary HCV infection. All of them successfully resolved the subsequent infection. Subjects’ demographics and clinical data are summarized in **Table 1**. Total PBMC samples from 7 subjects collected at 4 distinct time points during reinfection were used for RNA extraction and subsequent RNA-sequencing (RNA-seq): Pre-reinfection (baseline), Early acute (earliest available time point post estimated date of reinfection; ~ 2–9 weeks), Late acute (~ 14–26 weeks) and long-term follow-up (~ 28–71 weeks) **(Fig 1A)**.

**Table 1:** Subjects’ Demographics and Clinical information for samples analyzed by RNA-seq.

Subject	Sex	Age at reinfection	Ethnicity	rs12979860 IFNL4 genotype	HCV genotype 1^st^/2^nd^ infection	Testing interval (days)^a^	Interval between infections (days)^b^	Estimated days post-reinfection		ALT (U/L)	AST (U/L)	HCV viremia
SR1	F	33	Caucasian	CC	3a	27	91	Pre-reinfection	-13	28	19	-
					1b –low level of 1a			Early Acute	14	26	25	+
								Late Acute	193	28	25	-
								Follow up	457	29	25	-
SR2	M	50	Caucasian	CC	NA	98	1036	Pre-reinfection	-44	28	18	-
					1			Early Acute	54	37	36	-
								Late Acute	109	55	70	-
								Follow up	236	25	21	-
SR3	M	31	Caucasian	CC	3a	105	2706	Pre-reinfection	-52	ND	ND	-
					1a – 1b			Early Acute	53	50	175	+
								Late Acute	115	39	28	+
								Follow up	199	28	19	-
SR4	M	42	Caucasian	CT	1a	91	235	Pre-reinfection	-45	ND	ND	-
					1			Early Acute	46	48	16	+
								Late Acute	122	27	15	-
								Follow up	405	34	14	-
SR5	M	50	Caucasian	CC	NA	112	720	Pre-reinfection	-56	ND	ND	-
					NA			Early Acute	64	ND	ND	+
								Late Acute	147	ND	ND	-
								Follow up	418	ND	ND	-
SR6	M	43	Caucasian	CT	NA	92	2447	Pre-reinfection	-128	ND	ND	-
					1			Early Acute	46	36	25	+
								Late Acute	131	ND	ND	-
								Follow up	495	ND	ND	-
SR7	M	34	Caucasian	CC	NA	68	NA	Pre-reinfection	-34	ND	ND	-
					1a			Early Acute	58	ND	ND	+
								Late Acute	147	ND	ND	+
SR8	M	45	Caucasian	NA	NA	90	NA	Pre-reinfection	-45	ND	ND	-
					1a			Early Acute	45	ND	ND	+
								Late Acute	212	ND	ND	+
								Follow up	698	ND	ND	-

^a^ The interval between the last negative and first positive RNA tests that were used to calculate the estimated date of reinfection (EDI).

^b^ The interval between the first negative test following the primary infection and the estimated date of reinfection. NA: Not available

We employed principal component analysis (PCA) using the top 500 most variable genes to visualize the variation in gene expression at the different time points tested **(Fig 1B)**. PCA shows a nearly complete separation of Early acute time points from those of pre-reinfection along the PC1 (19.2%) and PC2 (12.2%) axes. These variations are reduced gradually, with the follow-up time points overlapping baseline with some variability within the PC1 and PC2 axes. Our previous PCA analysis of longitudinal RNA-seq samples collected during primary HCV infections demonstrated a clear separation of viremia positive time points [12]. Reinfection samples, however, did not completely segregate based on their viremia status **(Fig 1B)**. Nevertheless, to facilitate comparison between transcriptomic signatures of primary infection *versus* HCV reinfection, a similar approach of stratifying the Early and Late acute time points based on viremia status positive (+) or negative (-), was used for subsequent transcriptomic analyses.

Next, we applied a paired linear model to compare each time point from all samples to their pre-reinfection baseline. A total of 599 genes were differentially expressed at the Early acute (+) time point (387 upregulated and 212 downregulated), while none were identified in other groups (FDR<0.10, **Fig 1C)**. In summary, the strongest host response detected at the transcriptomic level occurs at the Early acute time point following HCV reinfection.

### Strong host response one day post-vaccine prime and boost in healthy subjects

Next, we used publicly available data to define whether similar transcriptome changes are induced by the T cell-based HCV vaccine strategy of ChAd3-NS prime followed by an MVA-NS boost in healthy subjects [18]. PBMC samples were collected at four time points post-vaccine prime (d0, d1, d4 and d7) and boost (d56, d57, d60 and d63) and transcriptome changes were evaluated by microarray analysis **(Fig 1D)**. PCA shows a complete separation of d1 and d57 from other prime and boost time points along the PC1 axis, accounting for 61.3% of the variability (**Fig 1E)**. We applied a paired linear model to compare each time point to its baseline, and a total of 1172 genes were differentially expressed at d1 (387 upregulated and 785 downregulated), while 3441 genes were differentially expressed at d57 (1948 upregulated and 1493 downregulated) (FDR<0.05, **Fig 1F)**. Thus, prominent changes were detected 24 h post-vaccination, with the MVA-NS boost eliciting slightly stronger transcriptomic changes but with a similar transcriptomic signature. These results also highlight a quick return to baseline as early as 4 days after vaccination.

### Enriched B cell signatures in Early acute reinfection samples as compared to primary infection

**Fig 2:**
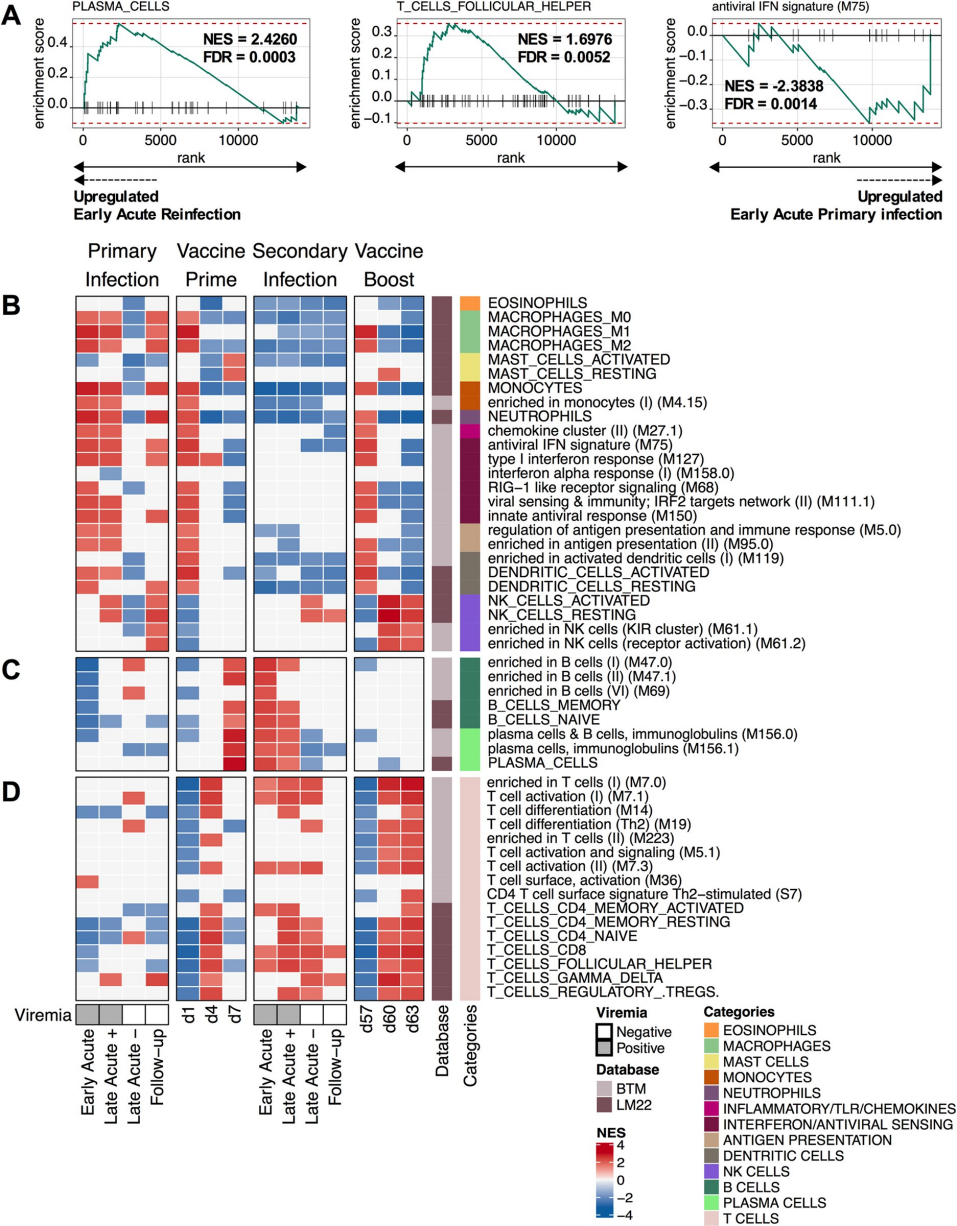
Upregulation of plasma cell modules at viremia positive time points during reinfection and their absence post-vaccine boosts. **(A)** Enrichment plots depicting differential enrichment of LM22 (PLASMA_CELLS and T_CELLS_FOLLICULAR_HELPER) and BTM (antiviral IFN signature (M75)) modules for the Early acute time point of the reinfection ([R]) relative to primary infection ([P]) using the following contrast: (R_EARLY-ACUTE_−R_PRE-REINFECTION_)–(P_EARLY-ACUTE_−P_PRE-INFECTION_). Primary infection data were obtained from (Rosenberg et al, 2018). Each gene forming the indicated module is represented by vertical black lines along the *x*-axis. The green line connects enrichment scores (ES) indicated by the *y*-axis and genes. Red-dashed lines indicate the maximum and minimum ES reached. False discovery rate (FDR) and normalized ES (NES) are presented with each enrichment plot. **(B-D)** Immune cell subsets (LM22 and BTM datasets indicated on sidebar) transcriptomic signature enrichment among the list of ranked differentially expressed genes at primary infection (Resolvers; n = 6), prime vaccine timepoints (n = 6), secondary infection (Resolvers; n = 7) and boost vaccine timepoints (n = 6). Pre-Infection times were used as a baseline for primary and secondary infections timepoints. d0 and d56 were used as a baseline for prime vaccine and boost vaccine time points, respectively. The *x*-axis indicates timepoints. The *y*-axis indicates immune subset enriched in at least one timepoint with a Benjamini−Hochberg-corrected gene set enrichment analysis (GSEA) *p* value < 0.05. Colors indicate the NES and show whether the immune subset was positively enriched (red) or negatively enriched (blue). Each module category is indicated on the color sidebar. Modules are further separated into three: **(B)** innate immune cells, **(C)** B cells and **(D)** T cells. The presence (Grey) or absence (White) of viral RNA is indicated below corresponding primary and secondary infection samples.

Our previous transcriptomic analysis of primary HCV infection ([P]) [12] and the analysis performed in the present reinfection study ([R], **Fig 1C**) demonstrated that the most pronounced transcriptome changes occur at the Early acute time point in both episodes. So, we sought to identify genes differentially increased in acute reinfection versus primary infection, normalized to their respective baselines, using the following contrast: (R_EARLY-ACUTE_−R_PRE-REINFECTION_)–(P_EARLY-ACUTE_−P_PRE-INFECTION_). The resulting ranked gene list was used to run gene set enrichment analysis (GSEA) using two databases specifically designed to study immune responses in peripheral blood: Leukocyte signature matrix (LM22, [20]) and blood transcriptome modules (BTM, [21]). Resolution of primary HCV infection was characterized by a strong upregulation of innate immune cells, inflammatory and interferon/antiviral-sensing signatures at Early acute. In contrast, the resolution of the secondary infection was characterized by a strong upregulation of B and T cell signatures **(Fig 2A and S1 Table)**.

Next, we ran GSEA for the reinfection episode using the following contrasts: (R_TIME-POINTS_−R_PRE-REINFECTION_). All LM22 and a representative subset of BTMs are presented as a heatmap in **Fig 2B–2D**, while all modules are presented in **S1 Table**. At the Early acute reinfection, GSEA showed global downregulation of modules associated with innate immunity **(Fig 2B).** In contrast, we observed an upregulation of modules associated with an adaptive immune response including: T_CELLS_CD4_MEMORY_ACTIVATED, T_CELLS_FOLLICULAR_HELPER, plasma cells and B cells, immunoglobulins (M156.0), PLASMA_CELLS and T_CELLS_CD8. Our results at the transcriptomic level show that, unlike the innate immunity-dominated signature present in acute primary infection, HCV reinfection is characterized by adaptive T and B cell signatures, consistent with induction of immune memory responses that result in shorter duration and magnitude of viremia during HCV reinfection, as previously described [5, 6]. It is important to note that, given the more rapid course of reinfection, we may have missed very early transient innate immune signatures. Indeed, clearance of heterologous HCV re-challenge in the chimpanzee model was associated with a higher hepatic expression of ISG, as well as type I and II interferons [22]. While T cell activation is maintained at Late Acute (-), B cell signatures were downregulated following HCV reinfection clearance **(Fig 2C–2D)**. To ensure that the observed humoral signatures are not a result of an outlier, we applied sample level enrichment analysis (SLEA) using leading edge genes of the R_EARLY-ACUTE_−R_PRE-REINFECTION_ contrast **(S1A–S1C Fig)**. All subjects, except SR2 and SR6 exhibited upregulation of B cell and plasma cell modules at Early acute as compared to pre-reinfection, with SR5 presenting the highest increase **(S1B Fig)**. In conclusion, the upregulation of modules associated with a humoral immune response observed at Early acute reinfection is a common transcriptomic signature.

### Strong T cell but weak B cell transcriptomic signatures post-HCV vaccine boost in healthy individuals

Next, we wanted to compare the transcriptomic changes induced by the T cell-based HCV vaccine prime (VP) and Boost (VB) with primary infection and reinfection signatures, respectively. The following contrasts were utilized for GSEA: (VP_TIME-POINTS_−VP_BASELINE (d0)_) and (VB _TIME-POINTS_−VB_BASELINE (d56)_) **(Fig 2B–2D, S1 Table)**. We observed at one day post-ChAd3 (d1) and post-MVA (d57) a shared upregulation of modules associated with innate immune cells, inflammatory and interferon/antiviral-sensing categories **(Fig 2B, S1 Table)**. These signatures rapidly waned and were followed by a transient or sustained upregulation of T cell modules at d4 and days 60–63, respectively **(Fig 2D).**

We also observed an upregulation of NK-cell related gene sets at d60 and d63 post-boost. Costanzo et al. had previously observed an up-regulation of genes associated with NK cells activity in healthy subjects 7 days after receiving an MVA vectored HIV-1 vaccine [23]. Their data and our observations (Fig 2B) support a model whereby vaccination using an MVA vector may increase NK cell functionality and cytotoxicity, ultimately potentially contributing to T cell priming, proliferation and memory T cell formation. Characterization of NK cells functionality longitudinally post-HCV vaccination using flow cytometry is however required to confirm this hypothesis.

At d7 post-prime, we observed an upregulation of B cell and plasma cell modules **(Fig 2C).** While the upregulation of plasma cell modules observed post-prime could potentially reflect an HCV-specific antibody response towards NS viral proteins, the absence of these signatures, that would indicate a recall response, post-boost suggests that this is not an HCV-specific response **(Fig 2C)**. Thus, the humoral response signatures observed at d7 may potentially reflect a response towards the ChAd vector or an increase in a pre-existing Adenovirus cross-reactive response. Indeed, high levels of ChAd3 neutralizing antibodies (nAbs) were detected post-prime in healthy subjects [24].

### HCV-specific antibodies in plasma increase at Early acute post-reinfection

Since we observed a strong plasma cell signature post-reinfection, we sought to evaluate the longitudinal polyclonal HCV-specific antibody responses by measuring relative levels of E2- and NS3-specific antibodies (IgG) in plasma by ELISA **(S2A Fig)**. We detected an E2-specific antibody response at pre-reinfection in 4 subjects (SR2, SR5, SR6 and SR8) against E2 from the H77 strain (genotype 1a; gt1a) and in 3 subjects (SR5, SR6 and SR8) against E2 from the J6 strain (gt2a). The anti-E2 antibody response increased significantly at the Early acute time point as compared to pre-reinfection, and rapidly decreased by the Late acute time points for all subjects except SR8 **(S2A Fig).** At pre-reinfection, plasma from 5 of 8 subjects (SR1, SR2, SR4, SR6 and SR8) contained antibodies against NS3 (gt1a). We detected a significant increase in anti-NS3 antibodies at Early acute as compared to pre-reinfection. This response was sustained at Late acute and decreased at Follow-up for all subjects except SR2 **(S2A Fig)**. Altogether, a strong anti-E2 and NS3 response was detected by ELISA at the Early acute time point following reinfection.

### HCV-specific antibodies in plasma neutralize genotype 1a but not genotypes 2a and 3a HCVpp

Next, we proceeded to evaluate the longitudinal nAb responses in plasma against HCV pseudoparticles (HCVpp) from genotypes 1a (H77), 2a (J6/JFH1), and 3a (S52). Herein, a neutralizing activity ≥50% was considered positive. For H77 HCVpp, neutralization was detected in three subjects (SR2, SR5 and SR8) at pre-reinfection **(S2B Fig).** Yet, such nAbs did not provide sterilizing immunity, *i*.*e*. did not prevent the establishment of HCV reinfection. The neutralizing activity in plasma increased significantly in 6 out of 8 subjects at Early acute. For SR3, SR5 and SR8, the neutralizing activity remained positive at the Late acute and follow-up time points. These results are consistent with a previous report which associated spontaneous clearance of HCV reinfections with the generation of nAbs against H77 [5]. All subjects neutralized J6/JFH1 (gt2a) and S52 (gt3a) HCVpp poorly for all time points. Only SR5 at Early acute neutralized S52 (gt3a) at 48%, denoting a transient cross-neutralizing capacity **(S2B Fig).** In summary, our data show that while E2-specific antibodies were detected against gt1a and gt2a, they were not capable of neutralizing non-gt1 HCVpp. Interestingly, all subjects were reinfected with either genotype 1a or 1b, which could explain poor cross-neutralizing (i.e. against gt2a and gt3a) capacities of plasma samples.

Next, we examined the correlation between the transcriptomic signature and antibody responses. The LM22 plasma cell module SLEA z-scores **(S1C Fig)** correlated positively with HCV-specific antibody responses and plasma neutralization activity but was only significant with the strongest antibody response detected (NS3) **(S2C Fig)**. This suggests that the humoral transcriptomic signatures observed during acute reinfection reflect an HCV-specific B cell response.

### Early activation and expansion of E2-specific memory B cells following HCV re-exposure

**Fig 3:**
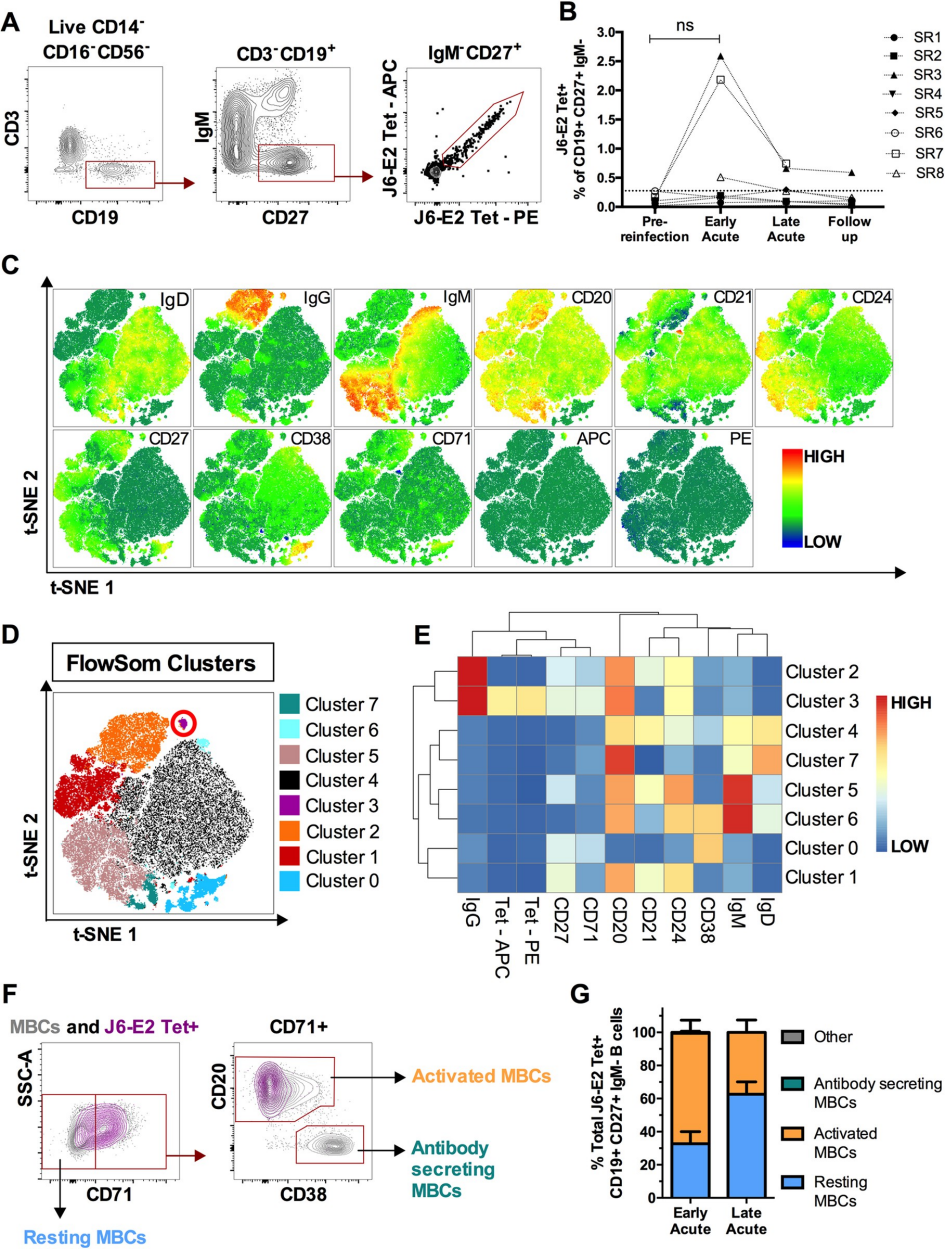
Activation and expansion of E2-specific MBCs following HCV reinfection. (**A**) Representative gating strategy for detection of class-switched, E2-specific MBCs (CD3^–^CD14^–^CD16^–^CD56^–^CD19^+^CD27^+^IgM^–^E2tet^+^) in total PBMCs. (**B**) Longitudinal frequencies of E2-specific (J6-E2 Tet^+^) MBCs at the indicated time points (see Fig 1A). Threshold of detection of E2-specific MBCs (dotted line) was 0.277% (mean detection + 2SD from 5 naive PWID). (**C**) Global t-SNE projection of CD3^–^CD14^–^CD16^–^CD56^–^CD19^+^ B cells from three subjects (SR3, SR7 and SR8) at the Early Acute time point, concatenated and overlaid. Each t-SNE projection reflects the expression of the indicated protein marker. (**D**) t-SNE projection of B cell clusters identified by FlowSOM clustering. (**E**) Heatmap shows mean fluorescence intensities (MFI) of each protein marker (columns), for each detected FlowSOM cluster (rows). (**E**) Representative gating strategy of resting MBCs (CD71^–^), activated MBCs (CD71^+^CD20^hi^CD38^int-lo^), antibody-secreting MBCs (CD71^+^CD20^lo^CD38^hi^) shown for the total MBCs population (grey contour plots), and HCV E2-specific MBCs (purple contour plots). (**F**) Phenotypes of E2-specific MBCs in resting (blue), activated (orange), or antibody-secreting (green) states (cells that did not meet these categories are designated as Other and shown in grey) at Early Acute and Late Acute time points (*n* = 3; SR3, SR7 and SR8). Significance was determined by Two-way repeated measure ANOVA with Tukey’s post hoc test (ns: not significant).

Next, we evaluated the longitudinal kinetic of E2-specific class-switched memory B cells (MBCs) using fluorescent J6-E2 tetramers **(Fig 3A)** [25]. Dual labeling was used to identify double-positive E2-specific MBCs (CD19^+^CD27^+^IgM^-^E2-tetramer^+^). The frequency of E2-specific MBCs peaked at Early acute in 3 out of 8 subjects (SR3, SR7 and SR8) and rapidly declined by Late acute **(Fig 3B)**. To better characterize the phenotype of these E2-specific MBCs, we concatenated the CD19^+^ B cell population of the three subjects with the highest tetramer^+^ frequency at Early acute and applied global high-dimensional mapping of the flow cytometric data **(Fig 3C)**. A t-distributed stochastic neighbor embedding (t-SNE) representation coupled with FlowSOM analysis allowed the identification of 8 B cell clusters **(Fig 3C–3E)**. According to the mean fluorescence intensity of each protein marker, cluster 3 delineates the E2-specific MBCs population. This cluster is characterized by the expression of a high level of CD20 and an intermediate one for the activation-related markers CD24 and CD71, suggesting recent antigenic stimulation **(Fig 3C–3E)**. The phenotype of E2-specific MBCs was confirmed through manual gating **(Fig 3F)**. At the Early acute time point, a resting phenotype (CD71^-^) was observed in 32.7% of E2-specific MBCs, while the majority (66.7%) had an activated phenotype (CD71^+^CD20^hi^CD38^int-lo^). Conversely, as their frequency decreased, the majority of E2-specific MBCs at Late acute had a dominant resting phenotype (62.6%) **(Fig 3G)**. These data show rapid expansion of E2-specific MBCs at Early acute followed by a decline in frequency and activation as the virus is cleared. We previously reported an increased frequency of E2-specific MBCs at Late acute, preceded by a peak of activated circulating T follicular helper (T_FH_) cells during primary infection in resolvers [26]. In the present study, we detected a rapid expansion of E2-specific MBCs at Early acute followed by a decline in their frequency and activation as the virus is cleared, consistent with a rapid and strong recall response. The role of T_FH_ during reinfection remains to be elucidated.

### Concerted increase in HCV-specific T and B cell responses at Early acute reinfection

**Fig 4:**
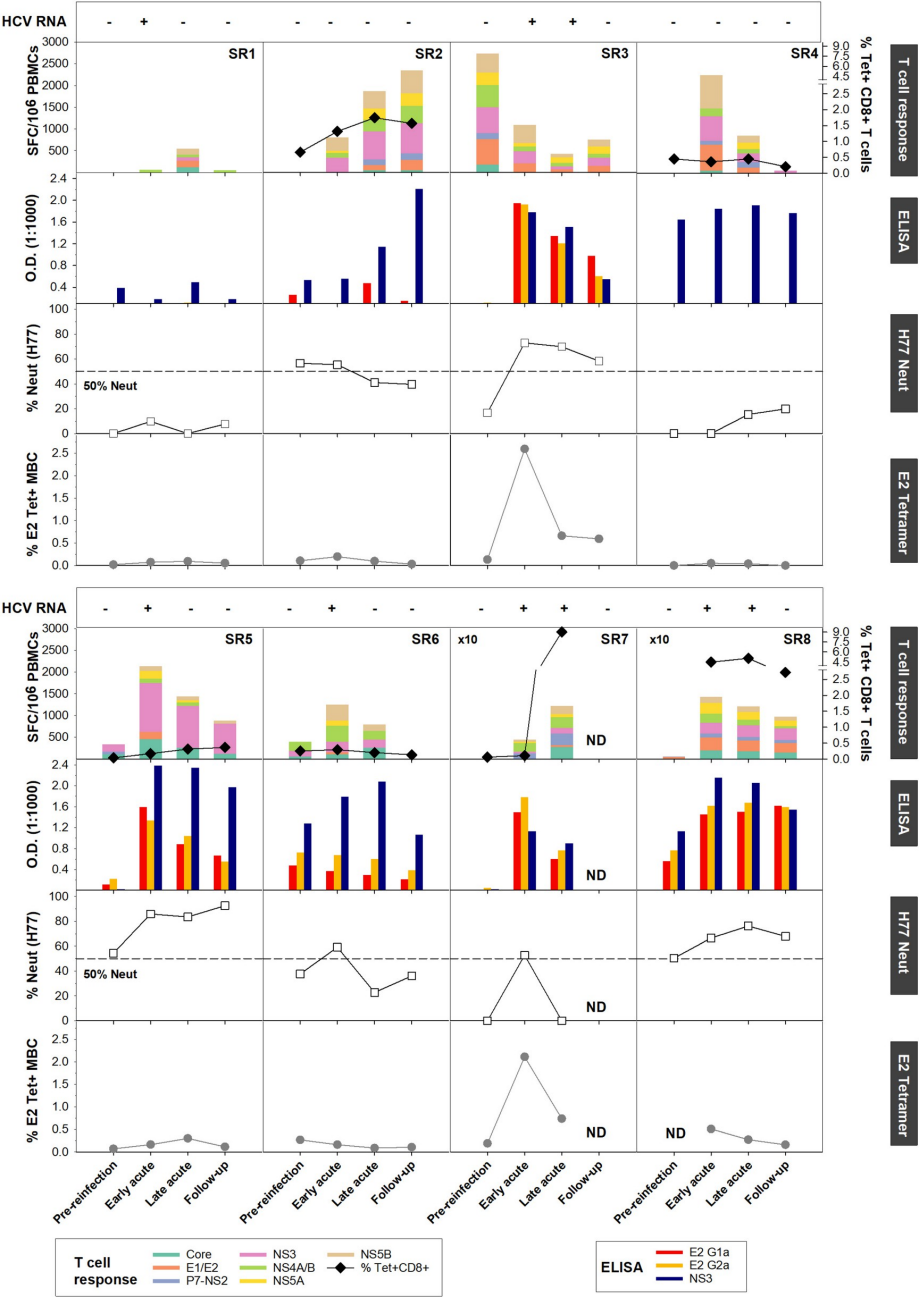
Concerted increase of HCV-specific T and B cell responses during acute reinfection. Results are presented per subjects with from top to bottom: **(HCV RNA)** Viremia status presented as viremia positive (+) and negative (-). **(Longitudinal IFN-γ ELISpot responses and MHC class I tetramer+ CD8+ T cells)**. PBMCs from the indicated time points were tested in an IFN-γ ELISpot assay against overlapping peptide pools representing the HCV H77 (SR2, SR3, SR5, SR6, SR7 and SR8) or J4 polyprotein (SR1 and SR4). The frequencies of IFN-γ spot forming cell (SFC) per million PBMCs from all subjects are presented in each top graph. When available, longitudinal MHC class I tetramer^+^ CD8^+^ T cells frequencies were assessed. The following tetramers were utilized: A1-1436 (SR2), A2-1073 (SR5), B7-41 (SR7 and SR8) and B27-2841 (SR4 and SR6). Subjects SR4 and SR6 ELISpot results were previously published in [6]. Pre-reinfection time-point different from the ones mentioned in Table 1 were used for subjects SR2 (-133) and SR3 (-815). **(ELISA)** Anti-E2 (gt1a and gt2a) and NS3 (Right) IgG responses in plasma measured by ELISA and represented as OD450–570. **(H77 HCVpp)** Plasma neutralizing activity against H77 HCVpp presented as percentage of neutralization relative to naïve donors. The dotted line delineates the 50% neutralization threshold. **(J6-E2 Tetramer)** Longitudinal J6-E2 Tetramer^+^ MBCs frequencies. No PBMC samples were available at pre-reinfection for subject SR8 to evaluate the frequency of J6-E2 Tetramer^+^ MBCs.

Next, we assessed the contribution of antibodies versus T cells in mediating viral clearance upon reinfection. The compiled data for each subject are summarized in Fig 4. We evaluated the magnitude and breadth of the HCV-specific T cell response longitudinally using an IFN-γ ELISpot assay against a panel of overlapping peptide pools representing either the HCV genotype 1a (H77) or 1b (J4) polyprotein **(Fig 4, top panels)**. In addition, in subjects for whom an MHC class I tetramer was available, we evaluated the longitudinal frequency of HCV-specific CD8^+^ T cells. Representative tetramer stainings are presented in **S3 Fig**. Consistent with our previous study [6], we observed an increase in the magnitude and breadth of the HCV-specific T cells measured by ELISpot in all subjects following reinfection, except SR3. We had previously demonstrated that such responses are mediated by both CD4^+^ and CD8^+^ T cells [6]. Indeed, subjects SR2, SR7 and SR8 displayed higher HCV-specific CD8^+^ T cells frequencies post-reinfection **(Fig 4, top panels).**

The majority of subjects studied (6 out of 8) demonstrated HCV-specific T and B cell responses but exhibited different patterns/kinetics. First, subjects SR1 and SR4 showed increased HCV-specific T cell responses but no E2-specific antibodies **(Fig 4, second panels)**, no neutralization capacity **(Fig 4, third panels)** and no expansion of E2-specific MBCs **(Fig 4, bottom panels)** suggesting that spontaneous clearance of HCV reinfection in these subjects was exclusively driven by T cells. Interestingly, SR1 displayed an upregulation of B cell signatures **(S1 Fig)**. This could reflect an HCV-specific antibody response targeting other NS proteins that we did not detect by measuring anti-NS3 or anti-E2 antibody responses. Second, subject SR2 demonstrated a weak E2-specific antibody response directed primarily towards the autologous E2 (gt1a) glycoprotein **(Fig 4, second panels)** and limited expansion of E2-specific MBCs **(Fig 4, bottom panels)**. Yet, these antibodies efficiently neutralized H77 (gt1a) HCVpp **(Fig 4, bottom panels)**, consistent with a focused antibody response driven by the autologous virus sequence. Third, subjects SR3, SR5, SR6 and SR8 exhibited a concomitant increase in T and B cell responses at Early acute. While only SR3 and SR8 had detectable E2-specific MBCs using J6-E2 tetramers **(Fig 4, bottom panels)**, all four subjects displayed increased neutralization capacity against H77 HCVpp at Early acute **(Fig 4, third panels)**. It is possible that expansions of E2-specific MBCs were missed in subjects SR5 and SR6 due to rapid HCV clearance. It is also possible that the E2-specific MBCs targeting autologous E2 of gt1a had limited reactivity/affinity towards the E2 gt2a tetramers. Finally, subject SR7 displayed an initial T cell response combined with a peak of E2-specific MBCs and antibody responses at Early Acute, followed by a decline in antibody responses at Late acute **(Fig 4)**. However, as the E2-specific antibody response and neutralizing capacity decreased, T cell responses peaked as the subject remained viremic at the Late acute time point. This suggests that the memory humoral and T cell responses may have not been very effective in controlling the acute reinfection in this subject early and that the delayed increase in breadth and magnitude of the T cell response may represent a *de novo* immune response, that efficiently cleared the infection.

In summary, the majority of subjects displayed increased E2-specific antibody responses and/or neutralizing capacities towards H77 HCVpp at Early acute together with HCV-specific T cells albeit to different magnitudes. These results are consistent with the upregulation of T and B cell modules **(Fig 2C–2D)** and suggest that resolution of HCV reinfection is characterized by concerted HCV-specific T and B cell responses.

### Concluding remarks

A better understanding of the correlates of protective immunity against HCV in real life setting is required for a better development of the next generation HCV vaccines. Here, we demonstrated that spontaneous resolution of HCV reinfection was characterized by a transient increase in E2-specific neutralizing antibody response. As previously reported [27], this antibody response rapidly waned after the control of viremia, consistent with the absence of plasma cell signatures at the HCV-RNA negative time points. Plasma samples mainly neutralized gt1a HCVpp, yet we cannot completely exclude that at the monoclonal level, some antibodies circulating at low level in blood could potentially neutralize heterologous strains. Indeed, Merat et al. previously identified broadly neutralizing Abs (bnAbs) at the monoclonal level, targeting the AR3 and AR4 regions of the E2 glycoprotein in subjects capable of clearing multiple HCV infections [28]. Such AR3-specific bnAbs were detected up to 5 years post-clearance of a primary infection, suggesting that such response could help prevent virus persistence upon reinfection.

We observed a concurrent increase in HCV-specific B and T cell responses at Early acute at the transcriptomic and functional levels. Although, in our study the concurrent impact of both immune responses on the infecting viral sequence is not known, our laboratory has previously shown that spontaneous resolution of HCV reinfections was associated with an expansion of polyfunctional clonotypes recruited from preexisting memory pools [6–8]. In addition, Kinchen et al. demonstrated that the capacity of nAbs to induce an immune pressure leading to mutations within the CD81 binding site of the E2 glycoprotein, eventually resulting in a loss of viral fitness [29]. It is therefore tempting to hypothesize that both these arms of adaptive immunity play a key role in HCV reinfection clearance. Interestingly, SR7 showed a different kinetic, displaying a strong nAbs response prior to the peak of T cell responses. This suggests that nAbs may have initially controlled the infection while the delayed peak of T cell response eventually led to HCV clearance. Further experiments are needed to confirm this hypothesis. Comparing real life HCV infections with vaccine-induced transcriptomic signatures in humans underscored the absence of plasma cell modules in post-MVA-NS vaccination as compared to acute reinfection. Because these viral vectored vaccines do not contain the glycoproteins E1 and E2, the absence of nAbs in response to this vaccine is expected. While different time frames between vaccine and natural infection time points may limit comparisons, the presence of an E2-specific antibody response in real-life cases of HCV reinfection and clearance provide an important rationale for a combination vaccine that is both T cell-based and bnAbs-inducing to prevent chronic HCV infection. AR3-specific bnAbs were previously detected in humans vaccinated with a recombinant E1E2 immunogen derived from a gt1a isolate (HCV-1), albeit at a low potency, suggesting that the induction of such bnAb responses by vaccination is possible [30, 31]. Future studies assessing samples from vaccination followed by infection in the Phase 2 trial of this vaccine regimen are warranted [10]. The transcriptomic signature associated with HCV re-exposure and clearance described in the present manuscript can be used as benchmarks to compare vaccine induced responses in that study and to evaluate novel HCV vaccines.

## Materials and methods

### Human ethics statement

All subjects provided written, informed consent prior to enrollment within the Montreal hepatitis C cohort (HEPCO) cohort. The ethics committee of the Centre hospitalier de l’Université de Montréal (CHUM) approved this study (Protocol SL05.014), which was conducted according to the ethical principles defined in the Declaration of Helsinki.

### Study subjects and samples

Study subjects are PWID recruited within the HEPCO cohort who spontaneously cleared an acute HCV primary infection. As previously described [19], clearance of a primary HCV infection was determined as two consecutive negative HCV RNA tests > 30 days apart (Cobas Ampliprep/Cobas TaqMan HCV Qualitative Test, version 2.0; Limit of detection: 15 IU/ml)). A subsequent HCV reinfection was defined as reappearance of HCV RNA. The median between the last negative and first positive HCV RNA test was used to determine the estimated date of reinfection (EDI). Study subjects were considered resolvers of reinfection, if HCV RNA was no longer detectable at 6 months post-EDI. Whole peripheral blood mononuclear cells (PBMCs) samples collected at four different time points were utilized: 1) Pre-reinfection baseline (Variable); 2) Early acute (2±9 weeks, mean 7 weeks); 3) Late acute (14±26 weeks, mean 19 weeks); and iv) Follow up (28±71 weeks, mean 41 weeks). Subjects’ demographics (e.g., age and sex), as well as key characteristics and estimated days post-reinfection are presented in Table 1.

### RNA-seq library preparation

Total RNA was extracted from thawed cryopreserved PBMC samples using the RNEasy Mini kit (Qiagen) according to manufacturer’s instructions. The RNA quality was assessed using the Agilent Bioanalyzer 2100 system (Agilent Technologies). All RNA samples displayed an RNA integrity numbers (RIN) score superior to 8. RNA samples were sent to the Genome Quebec Innovation Center for RNA-seq libraries preparation using the NEB mRNA stranded library preparation. Libraries were grouped by patient, in order to lessen the batch effect, and sequenced in multiplex on the illumina HiSeq4000 SR100 to generate 100 bp single-end reads (range 2.5x10^7^ ± 6x10^7^ total reads per library). Reads were obtained already aligned to the human genome (hg19). Read counts per gene were determined using HTSeq-count (v0.6.1p1) [32].

### Gene expression analysis

All the following analyses were performed within the R statistical framework (version 3.6.2) [33, 34] and Bioconductor [34], using reinfection samples, as well as primary HCV infection RNA-seq data from our previous study [12]. Principal component analysis (PCA) was performed as previously described [12] using the top 500 most variable genes across all resolver samples. Prior to normalization using the trimmed mean of M values (TMM) method from the edgeR package [35], genes with low expression values were removed. Genes with more than one read count per million (cpm) in at least three samples were included in the following analysis (15422 genes in total). Counts were then transformed to log2 CPM using the voom function [36] of the limma package [37]. For each HCV infection (primary [P] and reinfection [R]), a paired linear model was fitted with a donor blocking factor in which we compared each time point to its baseline. The resulting lists of pre-ranked genes given by *LIMMA* moderated *t*-statistic were used to perform gene set enrichment analysis (GSEA) [38], using the fgsea function from the fgsea package [39]. The leukocyte gene signature matrix (LM22) [20], as well as blood transcription modules [40] were used as gene sets for GSEA. Results were corrected for multiple testing using the Benjamini and Hochberg method [41]. Heatmaps were generated using the complex heatmaps package [42]. Sample level enrichment analysis (SLEA) was used to summarize the transcriptional level of each gene set and the sample level. SLEA compares the mean expression of genes within each module to the mean expression of 1000 randomly generated modules of the same size. The resulting *z*-score reflects the direction of the pathway in a sample, with a *z*-score > 0 indicating an upregulation and a *z*-score < 0, a down-regulation of the gene module.

### HCV vaccine transcriptomic signature

Microarray data for PBMC responses to the HCV vaccine (ChAd3-NSmut prime / MVA-NSmut boost) was graciously provided by Dr. Eleanor Barnes [18]. PCA, differential expression, and GSEA analyses were performed similarly to reinfection samples.

### Tetramers

Biotinylated E2 monomers were synthesized by the laboratory of Dr. Arash Grakoui as previously described [25]. Tetramers were prepared by adding phycoerythrin (PE)- (Sigma-Aldrich) or allophycocyanin (APC)-labeled ExtrAvidin (Thermofisher), 5 times using 7.5 μl with 10 min incubation at room temperature each time. Tetramer staining was performed using 0.5 μL of Tetramer-PE and 0.5 μL of Tetramer-APC per sample. The MHC class I monomers were synthesized by either the National Immune Monitoring Laboratory (Montréal, QC, Canada) or the NIH Tetramer Core Facility (Emory University, Atlanta, GA) and were as follows: HLA-B7 restricted HCV Core peptide aa 41–49 (GPRLGVRAT), HLA-A1 restricted HCV NS3 peptide amino acids (aa) 1436–1444 (ATDALMTGY) [A1/NS3-1436], HLA-A2 restricted HCV NS3 peptide aa 1073–1081 (CINGVCWTV) [A2/NS3-1073], HLA-B27 restricted HCV peptide NS5B peptide aa 2841–2849 (ARMILMTHF) [B27/NS5B-2841]. Tetramers were prepared by adding 10 μl of phycoerythrin (PE)-labeled ExtrAvidin (Sigma, St. Louis, MO) five times, with 10 min incubation at room temperature after each addition.

### Flow cytometry

Cryopreserved PBMCs were thawed and then washed with FACS buffer (PBS 1X, 1% FBS [Sigma-Aldrich], 0.01% sodium azide [Thermo Fisher Scientific]). Tetramer staining was performed for 30 minutes at room temperature (RT). Cells were then rewashed with FACS buffer and stained for 30 minutes at 4°C with surface marker and LIVE/DEAD fixable aqua dead cell stain kit (Thermo Fisher Scientific) to identify live cells. Directly conjugated monoclonal antibodies against the following cell-surface markers were used: CD3 APC-H7 (clone SK7), CD3–BUV496 (clone UCHT1), CD14 V500 (clone M5E2), CD4–BUV737 (clone SK3), CD8–BV786 (clone RPA-T8), CD16 V500 (clone 3G8), CD19 BUV496 (clone SJ25C1), CD21-BUV737 (clone B-ly4), CD24-BV605 (clone ML5), CD27 APC-R700 (clone M-T271), CD38 BUV395 (clone HB7), CD56 BV510 (clone NCAM16.2), IgD-PE-Cy7 (clone IA6-2), IgM BB515 (clone G20-127), all from BD Biosciences, San Diego, CA. IgG-BV421 (clone M1310G05), CD20 BV785 (clone 2H7), CD71 PerCP-cy5.5 (clone CY1G4) were from BioLegend, San Diego, CA. Cells were washed again and permeabilized using FoxP3 fixation and permeabilization buffer (Thermofisher Scientific) for 30 minutes at 4°C. Intracellular staining was done for 30 minutes at 4°C. Prior to FACS analysis, cells were fixed using 1% paraformaldehyde (PFA, Sigma-Aldrich). Multiparameter flow cytometry was performed at the flow cytometry core of the CRCHUM using a BD LSRFortessa instrument equipped with five lasers (UV [355 nm], violet [450 nm], blue [488 nm], yellow-green [561nm] and red [640 nm]) and the FACSDiva version 8.0.1 (BD Biosciences). FlowJo (version 10.0.8 for Mac; Tree Star, Inc.) was used to analyze FCS data files.

### ELISA assays

ELISA assays were performed as previously described [26] using 0.5 μg/mL of either in-house generated H77 E2 (genotype 1a) and J6/JFH1 E2 (genotype 2a) glycoproteins, or H77 NS3 protein (Raybiotech, recombinant NS3 (genotype 1a) amino acids 1192 to 1459). Plasma samples were diluted starting at 1:250 with 4-fold dilutions up to 1:16,000. All assays were performed in duplicates. Only the 1:1000 dilution results were shown in this study. The optical density (O.D.) was calculated by subtracting the mean absorbance at 450nm with the mean absorbance at 570nm.

### Neutralizing antibodies assays

HCV pseudotyped viruses (HCVpp) expressing a luciferase reporter alongside with the HCV glycoproteins derived from either genotype 1a (H77), genotype 2a (J6/JFH1) or genotype 3a (S52) were generated as previously described [26]. Neutralization assays were also performed as previously described [26]. Briefly, one day prior to infection human, hepatoma Huh7.5 cells were plated on collagen-coated 96-well plates (Corning). After complement inactivation, plasma samples were diluted (1:25) and mixed with H77, J6/JFH1 or S52 E1/E2 HCVpp for 1hour at 37°C, then added to Huh 7.5 cells. After 16–18 hours, the plasma-HCVpp inoculum was replaced with fresh culture media. After 48 hours, cells were processed using the Renilla Luciferase Assay Substrate (1X, Promega). Luminescence was then measured using a Synergy H1 Microplate Reader (Biotek Instruments). The neutralization activity was calculated using the following equation: % neutralization = (HCVpp _infection time point_—HCVpp _mock_) / HCVpp _mock_ ×100, where HCVpp _infection time point_ represents the luciferase activity of transduced cells in the presence of subject’s plasma and HCVpp _mock_ represents the luciferase activity of transduced cells in absence of any antibody. Three independent experiments were performed for all neutralization assays.

### Enzyme-linked immunospot (ELISpot) assay

Interferon-**γ** (IFN-**γ**) enzyme-linked immunospot (ELISpot) assay was performed against 11 pools of overlapping peptides representing the HCV genotype 1a (H77) or the genotype 1b (J4) polyproteins as previously described (Abdel-Hakeem and Shoukry, 2014) or using the Human IFN-gamma ELISpotPRO kit (ALP) from MabTech Inc (Cederlane, Ontario, Canada). Peptides were obtained from the Biodefense and Emerging Infections Research Resources Repository (BEI Resources, Manassas, VA, USA). All assays were performed in duplicates directly *ex vivo* on 2×10^5^ thawed PBMCs. For each peptide pool, spot forming cells (SFCs) were calculated as the mean number of spots in test wells minus the number of spots in negative control wells and normalized to SFC/10^6^ PBMCs. Subjects SR4 and SR6 ELISpot results were previously published in [6]. Pre-reinfection time-point different from the ones mentioned in Table 1 were used for subjects SR2 (-133) and SR3 (-815).

### Statistics

Statistical analyses were performed using either SigmaPlot (Systat Software), Prism versions 6.0 (GraphPad) or R for correlation analyses using the corrplot package. Details of tests are provided in each figure legend. Repeated measure ANOVA with Tukey’s post hoc test was used for longitudinal data. For all statistical tests, P values less than 0.05 were considered significant.

### RNA-seq data

All new gene expression data are deposited in the NCBI Gene Expression Omnibus (GEO) Accession number (GSE206397). Analysis codes can be found at https://github.com/shoukryLab.

## Supporting information

S1 Table LM22 and BTMs modules differentially enriched during HCV infections in PWID and post HCV vaccine prime and boost in healthy subjects. (XLSX)

S1 Fig Early acute reinfection transcriptional profile is enriched in B and T cells signatures. **(A-C)** Heatmaps displaying the sample level enrichment analysis (SLEA) *z*-score of each of the LM22 and BTMs modules using leading edge genes of the Early acute versus pre-reinfection contrast among all reinfected subjects. Rows represent individual modules from either LM22 (light blue) or BTMs (grey). Each module category is indicated on the colored sidebar. Modules are further divided into: **(A)** innate immune cells, **(B)** B cells and **(C)** T cells. Colors indicate the SLEA *z*-score and show whether the immune subset is upregulated (SLEA *z*-score > 0; red) or downregulated (SLEA *z*-score < 0; blue) in that sample. Columns represent individual subjects’ samples at the indicated time points. Detectable versus non detectable viremia is indicated in light grey and white boxes, respectively, all along the bottom of the heatmap.
(TIFF)

S2 Fig HCV-specific antibodies in the plasma of spontaneous resolvers increase and neutralize H77 HCVpp. (**A**) Longitudinal anti-E2 (Left and Middle panels) and NS3 (Right) IgG responses in plasma measured by ELISA and represented as OD450–570 for each subject (see Fig 1A for time point definitions). HCV antigens are indicated on top of each graph. Each symbol represents one subject (*n* = 8). The genotype of each infection is indicated next to each subject symbol as (primary infection | reinfection). (**B**) Longitudinal plasma neutralizing activity from all subjects against H77 HCVpp, J6/JFH1 HCVpp, and S52 HCVpp at 1:100 dilution, presented as percentage of neutralization relative to HCV-naive donor controls (n = 4). The dotted line delineates the 50% neutralization threshold. Results are presented as the mean of 3 independent experiments. For (A) and (B) repeated measure ANOVA with Tukey’s post hoc test was used. *P < 0.05; **P < 0.01. (**C**) Spearman correlations of all pairwise combinations of the designated parameters at the early acute time point. The intensity of the color and size of the circles reflect the values of corresponding correlations coefficients. Positive correlations are displayed in orange while negative correlations are displayed in purple.
(TIFF)

S3 Fig Representative gating strategy for identifying HCV-specific CD8+ T cells using MHC class I tetramers. Representative staining from the Late acute time point for all subjects studied are presented. Numbers in the upper right quadrant represent % Tetramer+CD8+CD3+ T cells.
(TIFF)
