# Supplementary figures and images for: Differential immune transcriptomic profiles between vaccinated and resolved HCV reinfected subjects
Source: PLoS Pathog. 2022 Nov 15;18(11):e1010968. doi: 10.1371/journal.ppat.1010968 (PMC9707775; doi:10.1371/journal.ppat.1010968)

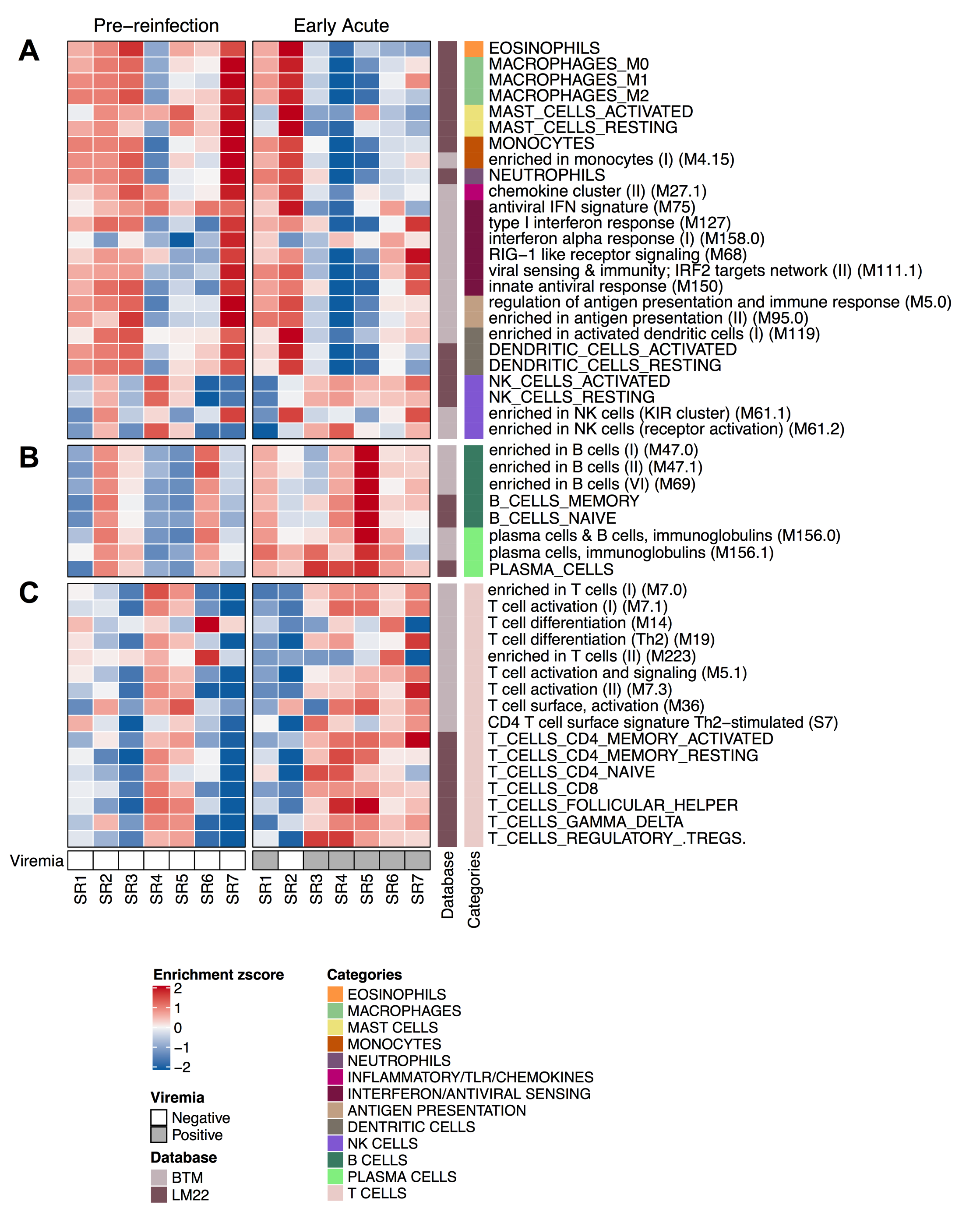

Supplement: S1 Fig — (A-C) Heatmaps displaying the sample level enrichment analysis (SLEA) z-score of each of the LM22 and BTMs modules using leading edge genes of the Early acute versus pre-reinfection contrast among all reinfected subjects. Rows represent individual modules from either LM22 (light blue) or BTMs (grey). Each module category is indicated on the colored sidebar. Modules are further divided into: (A) innate immune cells, (B) B cells and (C) T cells. Colors indicate the SLEA z-score and show whether the immune subset is upregulated (SLEA z-score > 0; red) or downregulated (SLEA z-score < 0; blue) in that sample. Columns represent individual subjects’ samples at the indicated time points. Detectable versus non detectable viremia is indicated in light grey and white boxes, respectively, all along the bottom of the heatmap. (TIFF) [file ppat.1010968.s002.tiff]

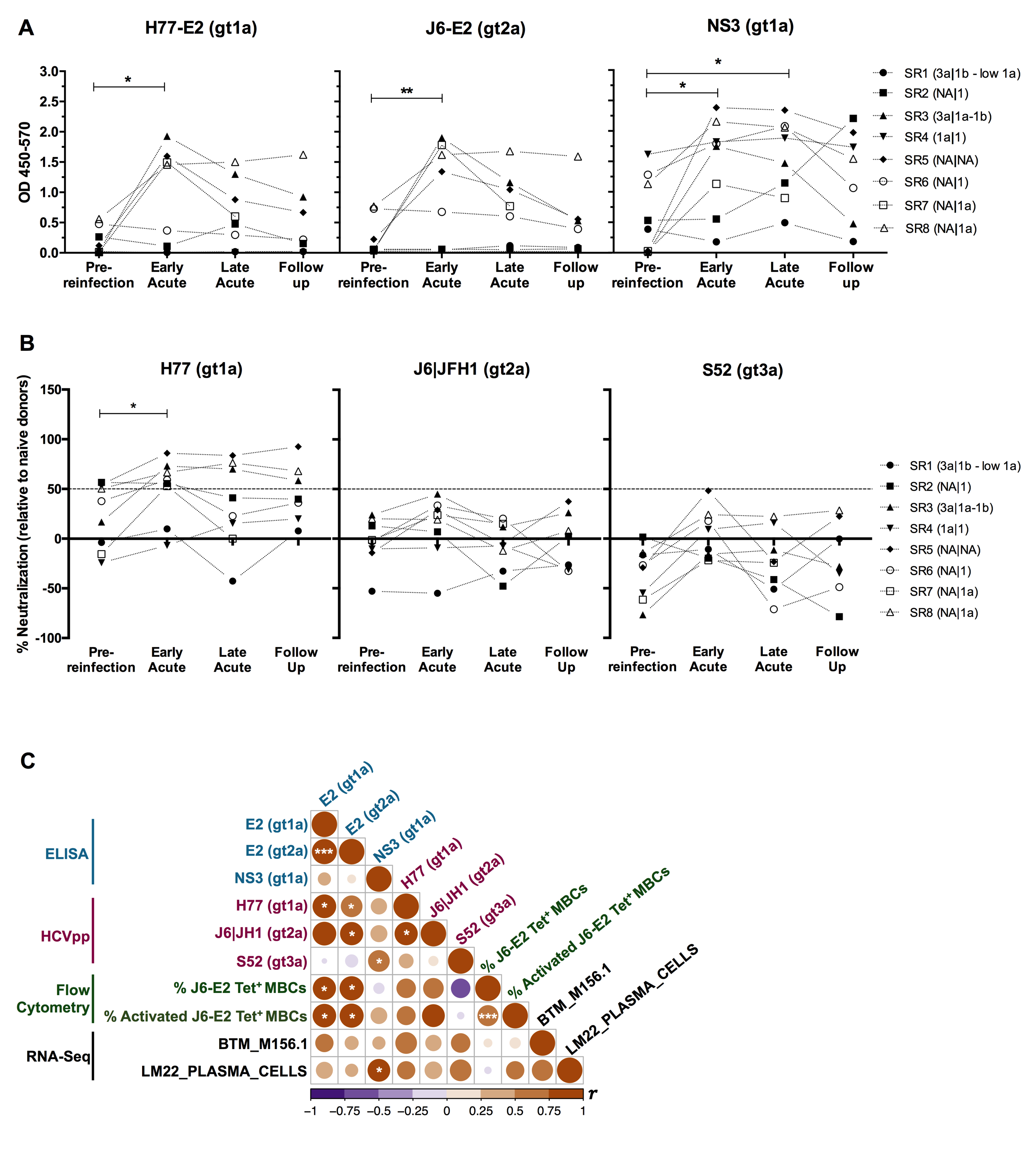

Supplement: S2 Fig — (A) Longitudinal anti-E2 (Left and Middle panels) and NS3 (Right) IgG responses in plasma measured by ELISA and represented as OD450–570 for each subject (see Fig 1A for time point definitions). HCV antigens are indicated on top of each graph. Each symbol represents one subject (n = 8). The genotype of each infection is indicated next to each subject symbol as (primary infection | reinfection). (B) Longitudinal plasma neutralizing activity from all subjects against H77 HCVpp, J6/JFH1 HCVpp, and S52 HCVpp at 1:100 dilution, presented as percentage of neutralization relative to HCV-naive donor controls (n = 4). The dotted line delineates the 50% neutralization threshold. Results are presented as the mean of 3 independent experiments. For (A) and (B) repeated measure ANOVA with Tukey’s post hoc test was used. *P < 0.05; **P < 0.01. (C) Spearman correlations of all pairwise combinations of the designated parameters at the early acute time point. The intensity of the color and size of the circles reflect the values of corresponding correlations coefficients. Positive correlations are displayed in orange while negative correlations are displayed in purple. (TIFF) [file ppat.1010968.s003.tiff]

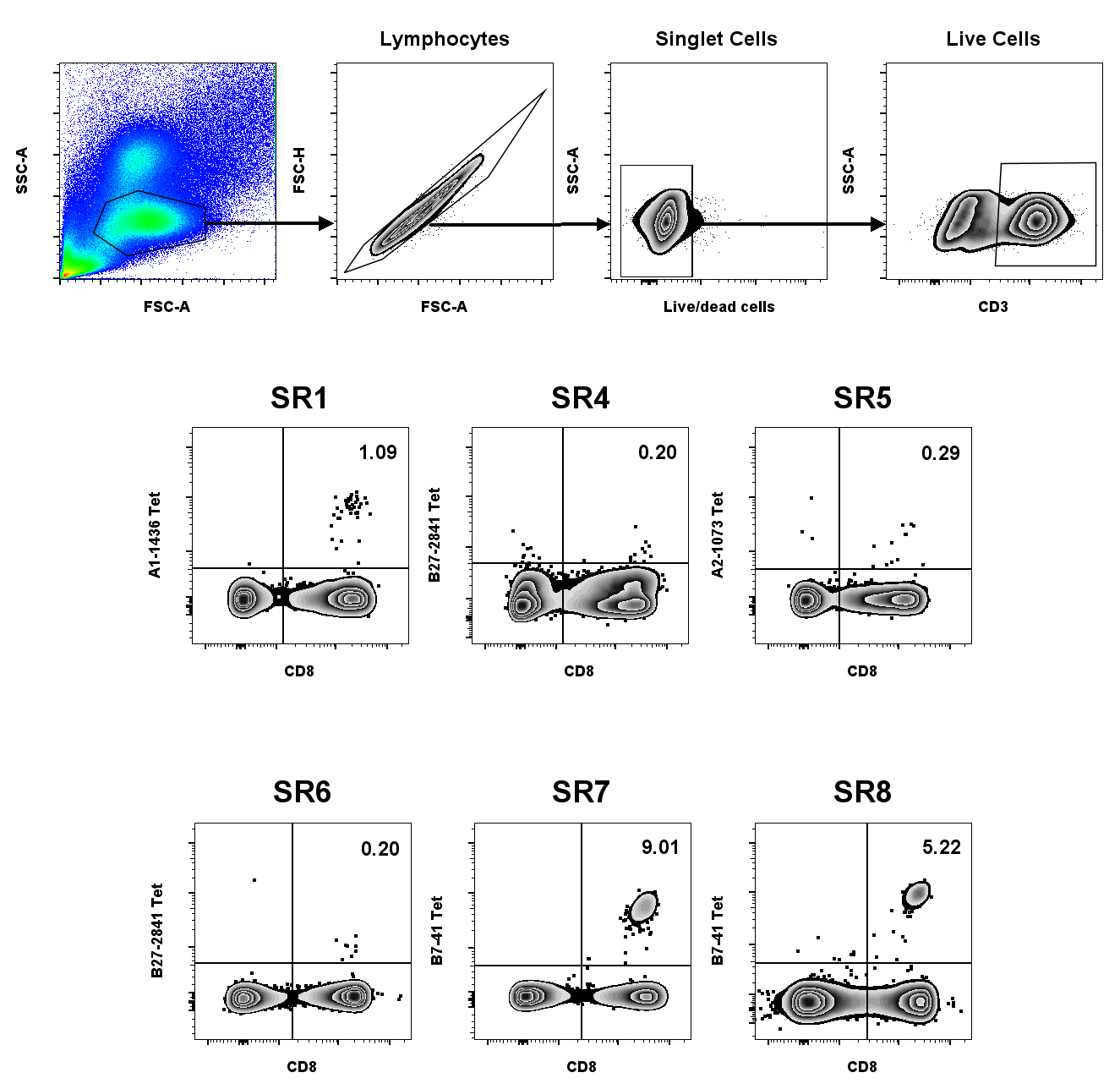

Supplement: S3 Fig — Representative staining from the Late acute time point for all subjects studied are presented. Numbers in the upper right quadrant represent % Tetramer+CD8+CD3+ T cells. (TIFF) [file ppat.1010968.s004.tiff]
